# Insights into the Flavor Profile of Yak Jerky from Different Muscles Based on Electronic Nose, Electronic Tongue, Gas Chromatography–Mass Spectrometry and Gas Chromatography–Ion Mobility Spectrometry

**DOI:** 10.3390/foods13182911

**Published:** 2024-09-14

**Authors:** Bingde Zhou, Xin Zhao, Luca Laghi, Xiaole Jiang, Junni Tang, Xin Du, Chenglin Zhu, Gianfranco Picone

**Affiliations:** 1 College of Food Science and Technology, Southwest Minzu University, Chengdu 610041, China; zhoubingde123@outlook.com (B.Z.); zhaoxinhh@outlook.com (X.Z.); junneytang@swun.edu.cn (J.T.); 2 Department of Agricultural and Food Sciences, University of Bologna, 47521 Cesena, Italy; l.laghi@unibo.it (L.L.); gianfranco.picone@unibo.it (G.P.); 3 College of Chemistry and Environment, Southwest Minzu University, Chengdu 610041, China; 4 College of Chemistry and Life Sciences, Chengdu Normal University, Chengdu 611130, China; 081056@ednu.edu.cn

**Keywords:** yak jerky, distinct muscles, aroma, taste, flavoromics, chemometrics

## Abstract

It is well known that different muscles of yak exhibit distinctive characteristics, such as muscle fibers and metabolomic profiles. We hypothesized that different muscles could alter the flavor profile of yak jerky. Therefore, the objective of this study was to investigate the differences in flavor profiles of yak jerky produced by *longissimus thoracis* (LT), *triceps brachii* (TB) and *biceps femoris* (BF) through electronic nose (E-nose), electronic tongue (E-tongue), gas chromatography–mass spectrometry (GC-MS) and gas chromatography–ion mobility spectrometry (GC-IMS). The results indicated that different muscles played an important role on the flavor profile of yak jerky. And E-nose and E-tongue could effectively discriminate between yak jerky produced by LT, TB and BF from aroma and taste points of view, respectively. In particular, the LT group exhibited significantly higher response values for ANS (sweetness) and NMS (umami) compared to the BF and TB groups. A total of 65 and 47 volatile compounds were characterized in yak jerky by GC-MS and GC-IMS, respectively. A principal component analysis (PCA) model and robust principal component analysis (rPCA) model could effectively discriminate between the aroma profiles of the LT, TB and BF groups. Ten molecules could be considered potential markers for yak jerky produced by different muscles, filtered based on the criteria of relative odor activity values (ROAV) > 1, *p* < 0.05, and VIP > 1, namely 1-octen-3-ol, eucalyptol, isovaleraldehyde, 3-carene, D-limonene, *γ*-terpinene, hexanal-D, hexanal-M, 3-hydroxy-2-butanone-M and ethyl formate. Sensory evaluation demonstrated that the yak jerky produced by LT exhibited superior quality in comparison to that produced by BF and TB, mainly pertaining to lower levels of tenderness and higher color, taste and aroma levels. This study could help to understand the specific contribution of different muscles to the aroma profile of yak jerky and provide a scientific basis for improving the quality of yak jerky.

## 1. Introduction

In recent years, the popularity of Yak (*Bos grunniens*) meat has increased among consumers, particularly for the western region of China, owing to its high protein and low fat levels, as well as the fact that it is naturally pollution-free [1,2]. Yak jerky, as a traditional yak meat product, is particularly favored by consumers for its high nutritional value, distinctive flavor and prolonged shelf life [3]. Currently, studies on yak jerky mainly focus on the impacts of processing techniques and/or different processing parameters on the quality of yak jerky. For example, Han et al. found that a significant increase in alcohol was produced by lipid oxidation in yak jerky with increasing natural drying time, and high-altitude yak jerky with a drying time of 75 d was preferred [4]. Ma et al. observed a notable elevation in protein carbonyl compounds, accompanied by a reduction in protein digestibility, in yak jerky samples subjected to prolonged processing times [5]. Fan et al. discovered that different drying methods could significantly affect the color, flavor and overall acceptability of yak jerky. Among the different types, microwave-dried yak jerky exhibited favorable color and flavor characteristics [6]. However, there is a lack of research on the effect of different muscles of yak on the flavor of yak jerky.

Different muscles of yak exhibit distinctive characteristics, particularly for Omic profiles. It has been found that the protein contents of six different muscles of yak were distinct, and *longissimus thoracis* (LT) contained the highest protein content [7]. Quantitative proteomic profiles showed that there were 88 expressed proteins significantly altered among different muscles of yak. Among them, structural proteins and heat shock proteins could be utilized as markers of tenderness to distinguish between different muscles of yak [8]. In addition, our previous study conducted a ^1^H-NMR-based metabolomic investigation of different muscles of yak, which demonstrated that LT, compared to *biceps femoris* (BF), pertained to significantly higher levels of formate and carnosine but significantly lower amounts of IMP, leucine, mannose, valine, inosine, isoleucine, threonine, alanine, phenylalanine and tyrosine [9].

Flavor is considered one of the most important features of yak jerky and plays an important role on consumer preference. The flavor of yak jerky is produced by a number of important biochemical reactions, namely protein hydrolysis and oxidation, lipid hydrolysis and muscle oxidation [4]. Furthermore, the addition of condiments, such as salt and spices, also has an impact on the flavor of the final product [10]. Until now, the flavor profile of yak jerky has remained unclear, and only a few papers have paid attention to yak jerky flavor investigation through GC-MS [4]. Due to the complexity of meat flavor, the combination of intelligent sensory and GC-based approaches has been widely applied to acquire a comprehensive overview of the flavor profile of meat products [11]. Kang et al. employed GC-MS and GC-IMS to ascertain distinctions in the principal volatile organic compounds (VOCs) present in the *longissimi dorsi* (LD), *triceps brachii* (TB) and BF of Tianzhu white yak. The results showed that Tianzhu white yak meat contains abundant metabolites, and the VOC levels in BF muscles were higher than those in LD and TB muscles [12]. Shen et al. found that a total of 198 volatile compounds were identified in shashliks, which confirmed that intelligent sensory technology combined with chemometrics is a promising approach to the flavor characterization of shashliks and other food matrices [13]. However, no studies have attempted to evaluate the flavor profiles of yak jerky via a combination of intelligent sensory techniques and GC-based approaches.

Therefore, we hypothesized that (i) the use of multiple approaches could more comprehensively outline the flavor profile of yak jerky than using a single technique; (ii) different muscles could alter the aroma and taste profiles of yak jerky; and (iii) E-nose and E-tongue could be considered promising tools to distinguish yak jerky produced by different muscles. To test such hypotheses, the aims of this study were to provide a characteristic profile of yak jerky flavor by employing a combination of intelligent sensory and GC-based methodologies and to systematically elucidate the differences in the aroma profiles of yak jerky derived from different muscles. This study constructs the flavor profiles of yak jerky, with an aim to apply flavor as a tool to discriminate between yak jerky produced by different muscles. Such attempts could demonstrate the potential usefulness of GC-based methodologies and intelligence sensory approaches for classification and future quality control purposes for yak products.

## 2. Materials and Methods

### 2.1. Sample Preparation

**Figure 1:**
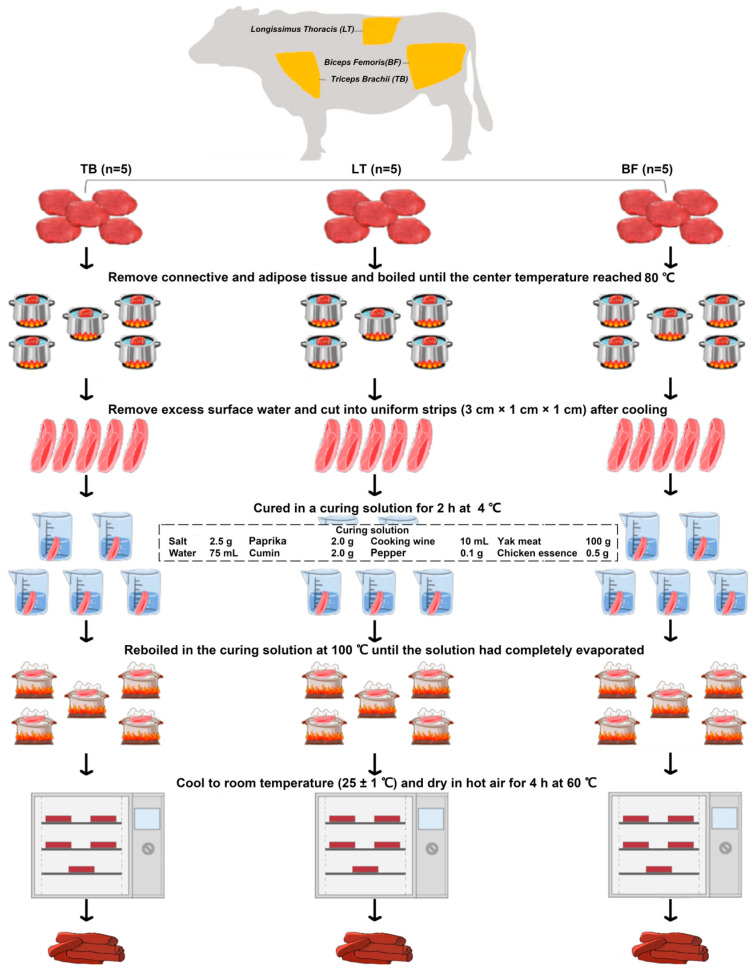
Sample preparation flowchart. *Longissimus thoracis* (LT), *triceps brachii* (TB) and *biceps femoris* (BF).

Different muscles (LT, TB and BF) of yak (4 years old, male) were purchased from a local supplier (Chengdu, China) and taken to the lab with ice and stored in a refrigerator (4 °C). According to the suggestions of Shi et al. [14], yak jerky was prepared through 6 steps, namely visible fatty and connective tissue removal, boiling, cutting, curing, re-boiling and drying, as depicted in Figure 1. In detail, after connective and adipose tissue removal, all the samples were boiled until the center temperature reached 80 °C. These samples were then cooled at room temperature and cut into uniform strips (3 cm × 1 cm × 1 cm) for curing. The curing process was conducted in a curing solution for 2 h at 4 °C. And then, samples were re-boiled in the curing solution at 100 °C until the solution had completely evaporated. Finally, the samples were cooled to room temperature, followed by drying in hot air for 4 h at 60 °C. The obtained samples were edible, complying with the microbial limit requirements of GB 2726-2016 and GB 29921-2021 [15,16]. Five samples were obtained for each group and stored in a refrigerator at −80 °C for the following set of analyses.

### 2.2. Analytical Methods

#### 2.2.1. E-Nose Analysis

With the equipment of 18 different metal oxide sensors, the Fox 4000 sensory E-nose analysis system (Alpha MOS, Toulouse, Haute-Garonne, France) was applied. A 0.25 g yak jerky sample was added to a headspace bottle (10 mL). Prior to analysis, all the samples were incubated at 70 °C for 5 min. The flavor substance was obtained with a specialized syringe and injected manually. The main parameters were as follows: equilibrium time pre-injection, 600 s; equilibrium temperature, 70 °C; and sample detection time, 120 s, as suggested by Zhao et al. [17]. The performance of each sensor is provided in Table S1. Every sample was tested ten times, and five stable values were used for the following data mining.

#### 2.2.2. E-Tongue Analysis

As suggested in the method used by Zhu et al. [18], *α*-ASTREE (Alpha MOS, Toulouse, Haute-Garonne, France) was performed for E-tongue analysis. A total of 7 sensors were equipped, mainly pertaining to umami (NMS), bitterness (SCS), sourness (AHS), sweetness (ANS), saltiness (CTS) and reference electrodes, namely PKS and CPS. 

Prior to analysis, a 20 g sample was chopped and mixed with deionized water (200 mL). The above solution was centrifuged at 2265× *g* and 4 °C for 10 min to obtain the aqueous phase (80 mL) for the following analysis. The deionized water was used as the washing solution, and the main parameters of the instrument were as follows: acquisition time, 120 s; stirring rate, 60 rpm; and washing time, 30 s. Every sample was tested ten times, and five stable values were used for the following data mining.

#### 2.2.3. GC-MS Analysis

The volatile components in the yak jerky were acquired and analyzed by HS-SPME (solid-phase microextraction fiber, 50/30 µm) and GC-MS (Thermo Fisher Scientific, Waltham, MA, USA) on a TG-WAXMS B column (30 m × 0.25 mm × 0.25 µm), with a Triplus auto-sampler (Thermo Fisher Scientific, Waltham, MA, USA). Following Luo et al. [19], 2 g of yak jerky sample and a 7.5 mL saturated salt solution were put in a sealed vial for 30 min at 50 °C. The aroma compounds were extracted by means of HS-SPME, followed by GC-MS analysis, and each sample was detected once. The injection port temperature was set to 270 °C and held for 15 min. The carrier gas was helium without a split mode. The initial oven temperature was set to 40 °C and held for 5 min. And then the temperature was increased at a rate of 5 °C/min to 220 °C and held for 5 min. Seventy electron volts of electron ionization and a 50–550 *m*/*z* scanning range were applied to obtain the mass spectrum. Volatile molecules were identified with the help of the NIST 11 library. Only values for the matching degree of molecules above 800 (maximum = 1000) were reported. a chromatographic peak area normalization method was used to calculate the relative concentration of each molecule.

#### 2.2.4. GC-IMS Analysis

GC-IMS (Flavorspec^®^, G.A.S. Instrument, Dortmund, North Rhine-Westphalia, Germany) was applied to analyze the volatile compounds in yak jerky, and each sample was detected once. A 0.25 g sample was equilibrated in vials (50 °C, 10 min), and 100 µL of the headspace sample was injected (65 °C) through an automatic headspace sampling system. The column of IMS was MXT-WAX (30 m × 0.53 mm × 1 µm) (Restek, Westport, CT, USA). The carrier gas (N_2_, 99.999% purity) flow rate was set to 150 mL/min, as reported in our previous paper [20]. The GC column flow rate was programmed as follows: 2 mL/min for 5 min, 10 mL/min for 10 min, 15 mL/min for 5 min, 50 mL/min for 10 min and 100 mL/min for 10 min. The retention index (RI) of each compound was calculated through external references, namely n-ketones C4–C9. Each sample was detected once, and the identification was applied through comparing their drift time and retention index with the GC-IMS library. Volatile compound quantification was based on the peak signal intensity. Using the Laboratory Analytical Viewer, Reporter, and Gallery Plot supported by the GC–IMS instrument, three-dimensional (3D) and two-dimensional (2D) topographic plots and gallery plots of the volatile compounds were constructed. 

#### 2.2.5. Sensory Evaluation

A 10-member sensory panel (5 males and 5 females) was uniformly trained following the methodology of Han et al. [4]. Each panelist provided informed consent, and the sensory evaluation received approval from the Ethics Committee of the Southwest Minzu University. Samples were presented randomly on disposable paper plates. Yak jerky was rated on four dimensions: aroma, color, tenderness and taste. A seven-point descriptive scale was used to measure these characteristics. The tenderness scale ranges from 1 (tough) to 7 (tender). Similarly, the color scale runs from 1 (dark brown) to 7 (saucy red). The aroma scale covers a spectrum from 1 (mild jerky odor, intense rancid odor) to 7 (intense jerky odor, mild rancid odor); the taste is rated on a scale from 1 (very bland) to 7 (strong taste). To cleanse their palate, the testers rinsed their mouths with drinking water after each evaluation.

### 2.3. Calculation of ROAVs

The relative odor activity values (ROAVs) of volatile compounds in yak jerky were calculated using the following formula [21]. Higher ROAVs of volatile components indicate a greater contribution to the sample odor. Volatile components with an *ROAV* ≥ 1 are considered key aroma components of the sample odor, while those with 0.1 ≤ *ROAV* < 1 are regarded as modifying components of the sample odor.

$$ROAV=100\times \frac{C_{A}}{C_{stan}}\times \frac{T_{stan}}{T_{A}}$$

*C_A_*: The relative content of volatile compounds in the yak jerky sample. 

*C_stan_*: The relative content of volatile compounds that contribute most to the overall aroma.

*T_stan_*: The odor threshold of volatile compounds that contribute most to the overall aroma.

*T_A_*: The odor threshold of volatile compounds in the yak jerky sample. 

### 2.4. Statistical Analysis

R language was applied for data mining. Before the following analyses, data normalization was applied according to Box and Cox [22]. ANOVA was employed to find out significant differences among groups with a cut-off value of *p* < 0.05. Following our previous study [23], robust principal component analysis (rPCA) models were established on the basis of the mean values of the response values of the E-tongue and E-nose sensors, as well as the molecules’ peak signal intensities, respectively. Referring to every rPCA model, a score plot and Pearson correlation plot were generated to elucidate an overview of the data and uncover the correlations between model components and variables. In addition, principal component analysis (PCA) and partial least-squares discriminant analysis (PLS-DA) were set up through an online data mining tool, namely MetaboAnalyst 5.0. Correlation analysis was performed using online tools (cloud.metware.cn, accessed on 15 May 2024). All experiments were conducted with five replicates per group.

## 3. Results

### 3.1. E-Nose and E-Tongue Analyses

**Figure 2:**
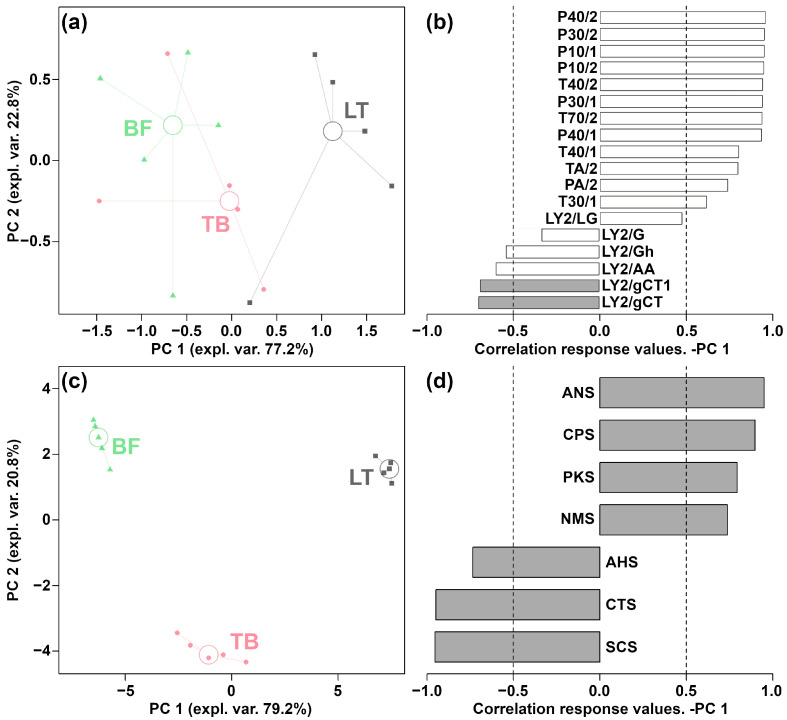
Score plot and loading plot of robust principal component analysis (rPCA) models based on electronic nose (E-nose) (**a**,**b**) and electronic tongue (E-tongue) (**c**,**d**) response data.

Following the method used by Zhang et al. [24], in order to differentiate between the overall aroma and taste features of yak jerky derived from different muscles, the response values of E-nose and E-tongue sensors were employed to construct rPCA models, respectively, as shown in Figure 2. The results indicated that E-nose and E-tongue could effectively discriminate between yak jerky produced by LT, TB and BF from aroma and taste points of view, respectively.

Focusing on the result of E-nose, as illustrated in Figure 2a, PC 1 accounted for 77.2% of the total variance observed in the entire sample set, which effectively encapsulates the aroma profile of yak jerky from different muscles. In detail, the LT group was mainly characterized by significantly higher response values for the LY2/LG, T30/1, P10/1, P10/2, P40/1, T70/2, PA/2, P30/1, P40/2, P30/2, T40/2, T40/1 and TA/2 sensors and by significantly lower response values for the LY2/Gh, LY2/G, LY2/gCT1, LY2/AA and LY2/gCT sensors, as shown in Figure 2b.

Similarly, as shown in Figure 2c, PC 1 accounts for 79.2% of the samples’ overall variability and effectively summarizes the differences among the three groups. The results of E-tongue showed that the LT group was mainly characterized by significantly higher response values for the ANS and NMS sensors and by significantly lower response values for the AHS, CTS and SCS sensors, as depicted in Figure 2d. 

### 3.2. GC-MS Analysis

**Figure 3:**
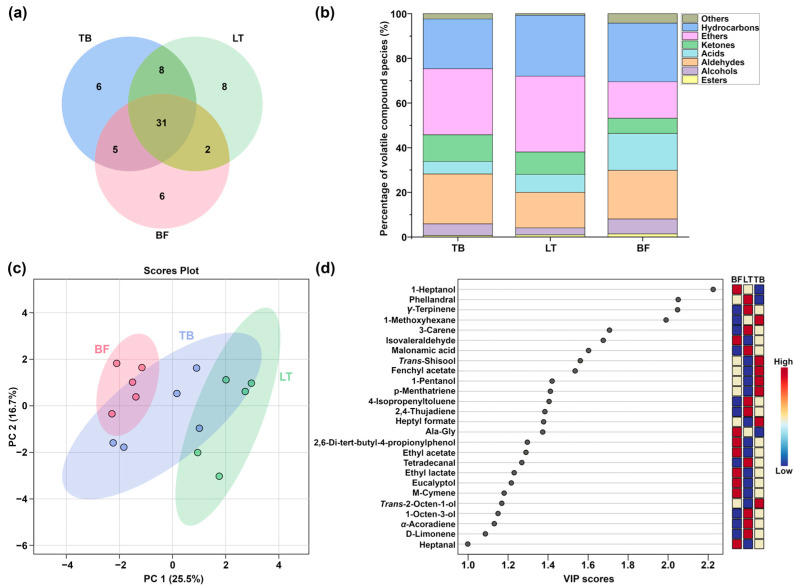
(**a**) Venn diagram plot of the number of volatile compounds in yak jerky from different muscles. (**b**) Bar plot of the percentage of volatile compound species in yak jerky from different muscles. (**c**) Principal component analysis (PCA) model based on volatile compounds in yak jerky from different muscles. (**d**) VIP score plots for the partial least-squares discriminant analysis (PLS-DA) model on volatile compounds in yak jerky from different muscles.

A total of 65 volatile compounds were characterized in yak jerky produced by TB, LT and BF by means of GC-MS, which could be sorted into eight species: aldehydes, alcohols, ketones, hydrocarbons, ethers, esters, acids and others. In detail, 49 volatile compounds (18 hydrocarbons, 10 alcohols, 7 aldehydes, 4 ketones, 4 ethers, 2 acids, 1 ester, 3 others) were characterized in yak jerky produced by LT. In terms of yak jerky produced by TB, it contained 49 volatile compounds (17 hydrocarbons, 10 alcohols, 9 aldehydes, 3 ketones, 4 ethers, 2 acids, 3 esters, 1 other). Moreover, 41 volatile compounds (13 hydrocarbons, 7 alcohols, 9 aldehydes, 4 ketones, 4 ethers, 1 acid, 3 esters, 2 others) were identified in yak jerky produced by BF. Detailed molecular information is reported in Table S2. As illustrated in Figure 3a, a total of 31 volatile compounds were identified across all groups. 

The percentage of volatile compound species varies among the groups, as shown in Figure 3b. The results from the PCA model (Figure 3c) demonstrated that the yak jerky samples produced by BF and LT were distinctly separated, while yak jerky produced by TB was located in between the other two. To further investigate the important volatile compounds in yak jerky from different muscles, VIP scores were calculated for the PLS-DA model. The Q^2^ and R^2^ values of the PLS-DA model are 0.652 and 0.971, respectively, indicating that the PLS-DA model is both reliable and free of overfitting. As illustrated in Figure 3d, the VIP values of 25 volatile compounds were above 1, which indicated their important roles in discriminating between yak jerky produced by different muscles.

### 3.3. GC-IMS Analysis

**Figure 4:**
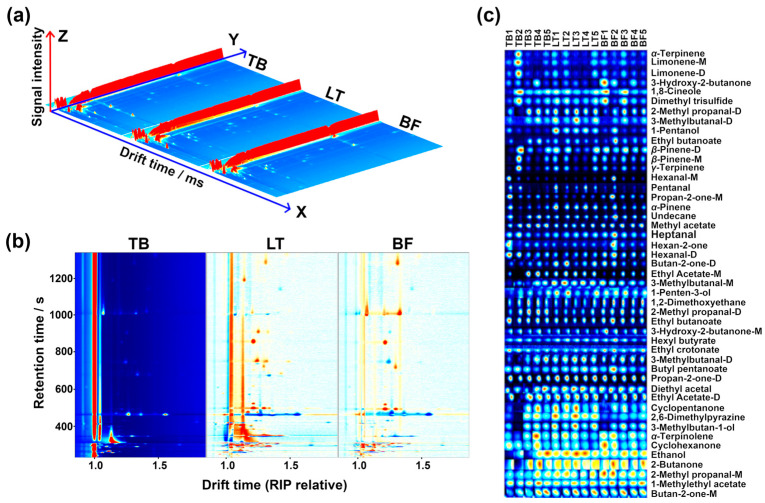
Gas chromatography–ion mobility spectrometry (GC-IMS) observation results of yak jerky from different muscles. (**a**) 3D topographic plot. (**b**) Subtraction plot, with spectra from TB group as a reference and the corresponding spectra from LT and BF groups represented as differences from TB group. (**c**) Gallery plots indicating the variations in volatile compounds’ concentrations among the four groups. Red and blue colors highlight over- and underexpressed components, respectively.

The aroma profile acquired by GC-IMS is illustrated in Figure 4. The 3D representation provides straightforward visual evidence that the samples from the three groups differ along the different portions of spectrum, as shown in Figure 4a. The subtraction plot revealed that a large number of peculiar compounds have an RT of between 400 and 1200 s, with ions showing drift times between 1.0 and 1.5 ms, as shown in Figure 4b. 

**Figure 5:**
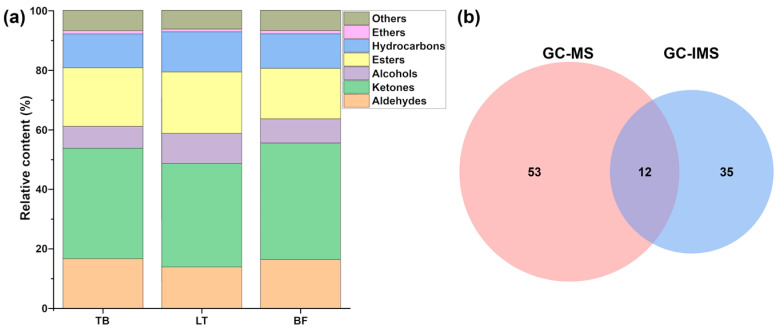
(**a**) Bar plot of the relative content of volatile compound species in yak jerky from different muscles characterized by GC-IMS. (**b**) Venn diagram plot of the number of volatile compounds characterized by GC-MS and GC-IMS.

In terms of molecule identification (Figure 4c), a total of 47 compounds were identified, pertaining to hydrocarbons (9), aldehydes (9), ketones (10), alcohols (6), esters (9), ether (1) and others (3). Their detailed information is reported in Table S3. Among them, the principal different volatile compounds present in the different groups were alcohols, aldehydes and esters, as shown in Figure 5a. Compared with GC-MS, only 12 volatile compounds (hexanal, heptanal, isovaleraldehyde, acetone, acetoin, ethanol, 1-pentanol, ethyl butanoate, ethyl acetate, *γ*-terpinene, *α*-terpinene, eucalyptol) were characterized by both techniques, while 35 and 53 unique volatile compounds were identified in GC-IMS and GC-MS, respectively, as illustrated in Figure 5b. 

**Figure 6:**
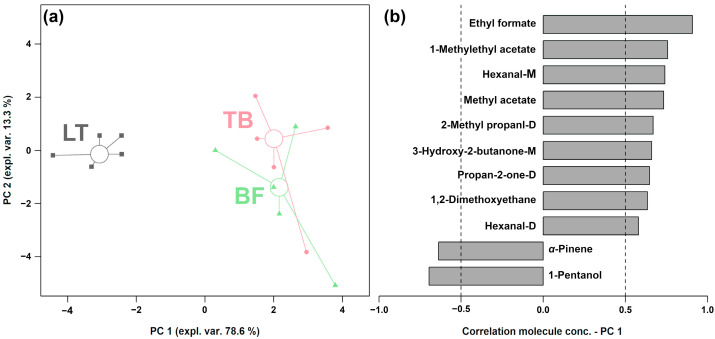
The rPCA model was established based on the relative content of GC-IMS differential volatile compounds. (**a**) The score plot shows the samples from the three groups as follows: squares (LT), circles (TB) and triangles (BF). The median of each group is represented by a wide and empty circle. (**b**) The loading plot illustrates the significant correlation between the molecule concentration and their importance along PC 1.

In line with Zhang et al. [25], an rPCA model was constructed using the concentration of these molecules to gain an insight into their trends. As shown Figure 6a, PC 1 accounts for 78.6% of the samples’ overall variability and effectively summarizes the differences among the three groups. Figure 6b reveals that the yak jerky produced by LT was characterized by significantly higher contents of *α*-pinene and 1-pentanol, while the yak jerky produced by TB and BF exhibited significantly higher contents of methyl acetate, 1-methylethyl acetate, ethyl formate, 2-methyl propanal-D, hexanal-M, hexanal-D, 3-hydroxy-2-butanone-M, propan-2-one-D and 1,2-dimethoxyethane.

### 3.4. ROAVs for Volatile Compounds

ROAVs can be employed as a screening tool for the identification of key characteristic volatile compounds in yak jerky from different muscles [21]. Among the molecules characterized by GC-MS and GC-IMS, 16 and 12 volatile compounds were identified in yak jerky, whose ROAVs were above 1, respectively (Table S4). Furthermore, a total of 10 molecules could be considered potential markers for yak jerky produced by different muscles, filtered based on the criteria of ROAV > 1, *p* < 0.05, and/or VIP > 1, namely 1-octen-3-ol, eucalyptol, isovaleraldehyde, 3-carene, D-limonene, *γ*-terpinene, hexanal-D, hexanal-M, 3-hydroxy-2-butanone-M and ethyl formate, as shown in Table 1.

### 3.5. Sensory Analysis

**Figure 7:**
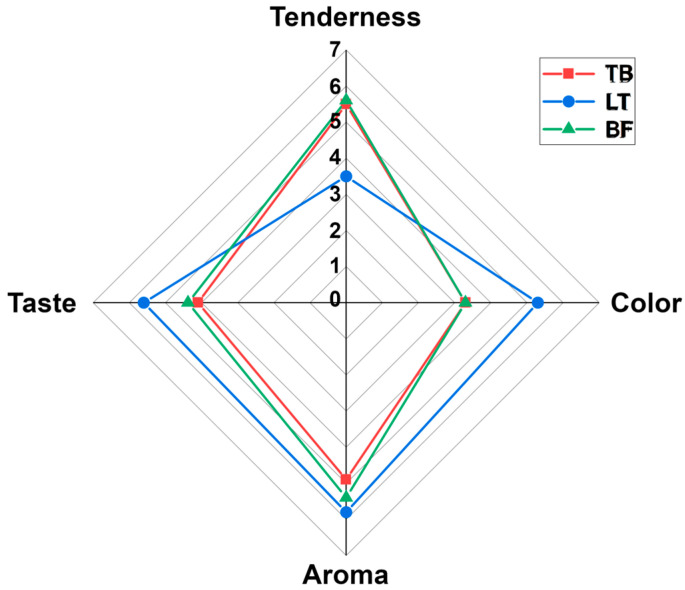
Radar chart for sensory evaluation of yak jerky from different muscles.

As illustrated in Figure 7, the color, aroma and taste of the yak jerky produced by LT were superior to those of the yak jerky produced by TB and BF. Conversely, the yak jerky produced by TB and BF exhibited superior tenderness compared to that produced by LT. The sensory evaluation analysis demonstrated that the yak jerky produced by LT exhibited superior qualities in comparison to the other two groups.

### 3.6. Correlation Analysis

**Figure 8:**
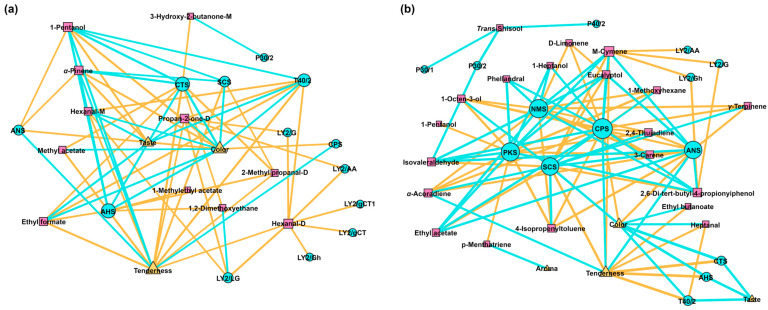
Correlation analysis of E-nose, E-tongue, sensory evaluation and volatile compounds quantified by GC-IMS (**a**) and GC-MS (**b**). The size of node is indicative of the number of substances that are significantly correlated with the substance in question. The blue circles represent the E-nose and E-tongue probes; the pink squares represent volatile compounds; and the yellow triangles represent sensory evaluation. In addition, the larger the node, the greater the number of substances with which it is significantly correlated. The thickness of line is representative of the size of the absolute value of the correlation between two substances. In this context, the thicker the line, the greater the absolute value of the correlation between the two substances.

In this study, we calculated Pearson correlations of E-nose, E-tongue, sensory evaluation and volatile compounds, retaining only strong (|cor| > 0.8) and statistically significant (*p* < 0.05) associations, as shown in Figure 8. Yak jerky taste was negatively correlated with the response values of AHS and CTS. The level of *p*-menthatriene was negatively correlated with yak jerky aroma. Both hexanal-D and hexanal-M were positively correlated with the LY2/LG and T40/2 of E-nose. 3-Hydroxy-2-butanone-M and isovaleraldehyde were negatively correlated with the P30/2 of E-nose. Ethyl formate was positively correlated with the T40/2 of E-nose.

## 4. Discussion

Yak meat, defined as “Green Food” by the Ministry of Agriculture of China, is low in fat and rich in many vitamins and amino acids [26]. Yak jerky is a popular product among consumers due to its distinctive flavor and ability to withstand storage. However, different muscles of yak are distinct, mainly pertaining to muscle tissue, protein content, fat content and flavor composition [7,12,27], which can consequently impact the quality of yak jerky. In this study, an attempt was made to comprehensively characterize the flavor and sensory profile of yak jerky from different muscles using E-nose, E-tongue and GC-based methods. 

E-nose and E-tongue could effectively differentiate between yak jerky produced by LT, TB and BF from aroma and taste points of view, respectively, which indicated that these two techniques could be considered effective potential fast approaches for discriminating between yak jerky from different muscles. However, in order to counterbalance the disadvantage of E-nose for the detection of specific aroma compounds, GC-based approaches were conducted to investigate the impact of different muscles on the aroma profile of yak jerky.

A total of 65 and 47 volatile compounds were characterized in yak jerky by GC-MS and GC-IMS, respectively. A total of 27 volatile compounds, including 1-nonanol, 1-octanol, 1-octen-3-ol, 1-pentanol, 3-carene, etc., were identified by GC-MS, which was in line with previous studies [6,28]. In addition, the yak jerky produced by different muscles exhibited distinct aroma profiles, which could be due to the fact that the rate of oxidation and the oxidation pathways might vary across different muscles, mainly pertaining to aldehydes [12]. It is worth noting that, in the present study, GC-MS was sensitive to alcohols and hydrocarbons, while GC-IMS was sensitive to aldehydes and esters. Therefore, the combination of GC-based approaches was able to supply a more systematic flavor profile of yak jerky than a single technique. Furthermore, the PCA and PLS-DA models were confirmed as efficacious methods for differentiating between the aromas of yak jerky produced from different muscles. 

Regarding hydrocarbons, aliphatic hydrocarbons are derived from the oxidation of lipids, while aromatic hydrocarbons are formed through the oxidation of branched aromatic acids [29]. Nevertheless, their higher thresholds restrict their capacity to influence the aroma of meat products. During the manufacture of yak jerky, the addition of spice agents could be considered the primary source of terpene production [30]. Among these compounds, *α*-pinene, *β*-pinene, D-limonene and *γ*-terpinene are derived from pepper and cumin [31,32]. 3-Carene has an olfactory profile that is reminiscent of pine, mint, citrus and camphor odors [33]. *α*-pinene, *β*-pinene and *γ*-terpinene had pine-like and bitter odors [34]. Moreover, *p*-cymene and D-limonene have citrus and carrot odors [35,36]. 

Aldehydes play substantial roles in the aroma profile of yak jerky and are mainly produced through amino acid degradation and fatty acid oxidation [37]. The results of our study indicated that the content of aldehydes was the highest in yak jerky, which was in line with a previous study [6]. Hexanal, a major metabolite of linoleic acid oxidation, possesses a distinctive grassy and fatty aroma [6]. The higher level of hexanal in yak jerky produced by the BF group could be linked to the higher content of unsaturated fatty acids in BF compared to LT and TB [38]. Oleic acid is one of the primary energy sources in active muscle areas such as BF and TB. The oxidation of oleic acid results in the formation of octanal and nonanal, which impart a fruity aroma profile [1], which result in the observed content differences among the groups. Heptanal is an oxidation product of arachidonic acid and possesses an aroma profile associated with roasted meat and nut [39]. It is worth noting that onanal, hexanal, pentanal and heptanal have been identified as odor-active compounds in cooked yak meat [29]. 2-Methyl propanal is a Strecker aldehyde that can be derived from the catabolism of branched-chain amino acids, with a malty and sweet odor [40]. Additionally, it has been identified as a factor contributing to discernible variations in the aroma of roasted beef [41]. Isovaleraldehyde and 3-methylbutyraldehyde can provide fruity, malty and chocolatey odors for yak jerky [42,43].

Ketones are primarily derived from Maillard reactions and lipid oxidation. Compared to aldehydes, their higher threshold value has a positive effect on the aroma of yak meat and harmonizes the overall aroma [1]. The formation of 3-hydroxy-2-butanone is associated with a Meladic reaction between cysteine and ribose [44]. A previous study has demonstrated that 3-hydroxy-2-butanone could provide the buttery odor of beef and related products [45]. The low threshold of 2-acetone contributes to the fatty odor of cooked meat. Additionally, 2-propanone has been identified as one of the main aroma constituents of cooked beef, with a relative percentage abundance of 71% [40].

Esters are synthesized through esterification reactions and transesterification reactions. Ethyl formate, 1-methylethyl acetate and methyl acetate are commonly found in meat products [46] and have been confirmed to have crucial positive effects on the aroma profiles of meat products [47]. Among these, ethyl formate can provide an aromatic, pineapple-like odor for yak meat products [48]. 

The primary source of alcohols is the degradation of linoleum in muscle tissue by lipoxygenase and peroxidase. However, alcohols possess high odor thresholds, which limits their impacts on the aroma of yak meat [1]. 1-Pentanol, also known as butyl methanol, is able to provide pleasant fruity and balsamic odors [49]. It has been considered one of the odor-active compounds present in steamed high-altitude yak meat [29]. The elevated concentration of 1-pentanol observed in yak jerky produced by LT may be associated with the enhanced muscle fatty acid oxidative activity of LT compared to the other muscles. 1-Heptanol has been identified as a key aroma compound in roast beef, exhibiting a floral and green aroma [50]. 1-Nonanol has a fatty and citrusy odor [51]. Fan et al. found that 1-octen-3-ol could be considered a key volatile compound in yak jerky with a mushroom aroma [6]. A significantly higher level of 1-octen-3-ol in yak jerky produced by LT could be linked to the higher levels of arachidonic and linoleic acids in LT [12], which could produce 1-octen-3-ol through degradation [52].

## 5. Conclusions

The present work attempted to obtain the flavor profile of yak jerky produced by LT, TB and BF by means of a combination of E-nose, E-tongue and GC-based approaches. The sensory evaluation demonstrated that the yak jerky produced by LT exhibited superior quality in comparison to that produced by BF and TB, mainly pertaining to lower levels of tenderness and better color, taste and aroma characteristics. Combined with E-tongue analysis, the better quality of the LT group could be partly attributed to higher levels of sweetness and umami compared to those of the BF and TB groups. More volatile compounds could be characterized by multiple approaches, mainly sourced from the production of yak jerky and the addition of spices. Taking advantage of univariate and multivariate analyses, 1-octen-3-ol, eucalyptol, isovaleraldehyde, 3-carene, D-limonene, *γ*-terpinene, hexanal-D, hexanal-M, 3-hydroxy-2-butanone-M and ethyl formate could be considered potential markers for the classification of yak jerky produced by different muscles. This study could provide a reference guide for constructing a comprehensive flavor profile of yak jerky and offer new insights into using a combination of analytical techniques to explore and differentiate between flavor profiles in yak products.

## Figures and Tables

**Table 1:** Relative odor activity values (ROAVs) of key volatile compounds in yak jerky from different muscles characterized by GC-MS and GC-IMS.

	Compound Name	Threshold (μg/kg)	ROAV		
			TB	LT	BF
GC-MS	1-Octen-3-ol	1.5	4.708299	9.998937	–
	Eucalyptol	3	–	–	5.961556
	Isovaleraldehyde	0.25	–	–	16.63372
	3-Carene	0.4	5.123714	14.24697	–
	D-Limonene	10	18.62289	45.15313	10.75404
	*γ*-Terpinene	260	1.425817	3.924652	0.791342
GC-IMS	Hexanal-D	5	1.831927	0.929906	1.673773
	Hexanal-M	5	3.779587	2.878009	3.201198
	3-Hydroxy-2-butanone-M	0.5	100	100	100
	Ethyl formate	5	1.707558	1.541432	1.534396

“–” means not detected.

## Data Availability

The original contributions presented in the study are included in the article/Supplementary Material, further inquiries can be directed to the corresponding authors.

## Supplementary Materials

The following supporting information can be downloaded at https://www.mdpi.com/article/10.3390/foods13182911/s1: Table S1. E-nose sensors and its corresponding representative sensitive compounds; Table S2. The relative content of volatile compounds in yak jerky from different muscles characterized by GC-MS (Mean ± SD); Table S3. The relative content of volatile compounds in yak jerky from different muscles characterized by GC-IMS (Mean ± SD); Table S4. ROAV of volatile compounds in yak jerky from different muscles characterized by GC-MS and GC-IMS.
