# Supplementary material for: Insights into the Flavor Profile of Yak Jerky from Different Muscles Based on Electronic Nose, Electronic Tongue, Gas Chromatography–Mass Spectrometry and Gas Chromatography–Ion Mobility Spectrometry
Source: Foods. 2024 Sep 14;13(18):2911. doi: 10.3390/foods13182911 (PMC11431100; doi:10.3390/foods13182911)
Supplement: Supplementary file 1 [file foods-13-02911-s001.zip › foods-3187258-supplementary.pdf]

Insights into the Flavor Profile of Yak Jerky from Different Muscles Based on Electronic Nose, Electronic Tongue, Gas Chromatography–Mass Spectrometry and Gas Chromatography–Ion Mobility Spectrometry

Bingde Zhou [1,†], Xin Zhao [1,†], Luca Laghi [2], Xiaole Jiang [3,*], Junni Tang [1], Xin Du [4], Chenglin Zhu [1,*] and Gianfranco Picone [2]

Supporting Materials

Table S1. E-nose sensors and its corresponding representative sensitive compounds

Sensors	Performance description
LY2/LG	Sensitive to oxidizing gas
LY2/G	Sensitive to ammonia, carbon monoxide
LY2/AA	Sensitive to ethanol
LY2/Gh	Sensitive to ammonia/organic amines
LY2/gCT1	Sensitive to hydrogen sulfide
LY2/gCT	Sensitive to propane/butane
T30/1	Sensitive to organic solvents
P10/1	Sensitive to hydrocarbons
P10/2	Sensitive to methane
P40/1	Sensitive to fluorine
T70/2	Sensitive to aromatic compounds
PA/2	Sensitive to ethanol, ammonia/organic amines
P30/1	Sensitive to polar compounds (ethanol)
P40/2	Sensitive to heteroatom/chloride/aldehydes

P30/2	Sensitive to alcohol
T40/2	Sensitive to aldehydes
T40/1	Sensitive to chlorinated compounds
TA/2	Sensitive to air quality

Table S2. The relative content of volatile compounds in yak jerky from different muscles characterized by GC-MS (Mean ± SD).

Compound	Cas	Molecular formula	RT	SI	Relative content		
					TB	LT	BF
**Esters**							
Ethyl butanoate	105-54-4	$C_6H_{12}O_2$	3.66	677	$1.73\times10^{-4} \pm 1.75\times10^{-4}$	ND	$2.99\times10^{-4} \pm 2.79\times10^{-4}$
Heptyl formate	112-23-2	$C_8H_{16}O_2$	9.65	887	$9.76\times10^{-4} \pm 1.24\times10^{-3}$	ND	ND
Fenchyl acetate	13851-11-1	$C_{12}H_{20}O_2$	10.41	806	$8.21\times10^{-4} \pm 7.21\times10^{-4}$	ND	ND
Ethyl acetate	141-78-6	$C_4H_8O_2$	2.37	637	ND	ND	$1.74\times10^{-3} \pm 1.46\times10^{-3}$
Vinyl hexanoate	3050-69-9	$C_8H_{14}O_2$	7.22	800	ND	$9.71\times10^{-4} \pm 8.56\times10^{-4}$	ND
Ethyl lactate	97-64-3	$C_5H_{10}O_3$	7.62	790	ND	ND	$2.39\times10^{-3} \pm 1.97\times10^{-3}$
**Alcohols**							
1-Heptanol	111-70-6	$C_7H_{16}O$	9.65	843	ND	$5.74\times10^{-4} \pm 4.81\times10^{-4}$	$9.99\times10^{-4} \pm 3.21\times10^{-4}$
1-Octanol	111-87-5	$C_8H_{18}O$	11.88	852	$3.80\times10^{-3} \pm 3.15\times10^{-3}$	$2.98\times10^{-3} \pm 1.19\times10^{-3}$	$3.98\times10^{-3} \pm 9.11\times10^{-4}$
Trans-Shisool	1413-55-4	$C_{10}H_{16}O$	12.14	843	$7.69\times10^{-3} \pm 3.99\times10^{-3}$	$7.02\times10^{-3} \pm 3.52\times10^{-3}$	$1.17\times10^{-2} \pm 4.17\times10^{-3}$
1-Nonanol	143-08-8	$C_9H_{20}O$	14.16	628	ND	$2.37\times10^{-4} \pm 2.25\times10^{-4}$	ND
Trans-2-Octen-1-ol	18409-17-1	$C_8H_{16}O$	13.20	743	$2.40\times10^{-4} \pm 2.05\times10^{-4}$	ND	ND
(-)-Terpinen-4-ol	20126-76-5	$C_{10}H_{18}O$	12.85	712	$7.44\times10^{-4} \pm 6.60\times10^{-4}$	$1.06\times10^{-3} \pm 4.45\times10^{-4}$	$5.88\times10^{-4} \pm 5.30\times10^{-4}$
(4-Prop-1-en-2-ylcyclohexyl) methanol	22451-48-5	$C_{10}H_{18}O$	18.80	602	$2.81\times10^{-4} \pm 2.95\times10^{-4}$	ND	ND
1-Octen-3-ol	3391-86-4	$C_8H_{16}O$	9.50	822	$3.28\times10^{-3} \pm 3.20\times10^{-3}$	$2.70\times10^{-3} \pm 1.75\times10^{-3}$	ND
Cuminol	536-60-7	$C_{10}H_{14}O$	23.48	873	$2.38\times10^{-3} \pm 4.59\times10^{-4}$	$2.09\times10^{-3} \pm 9.42\times10^{-4}$	$2.56\times10^{-3} \pm 7.83\times10^{-4}$

Ethanol	64-17-5	C_2H_6O	2.76	905	$5.12\times10^{-2} \pm 3.75\times10^{-2}$	$3.28\times10^{-2} \pm 1.37\times10^{-2}$	$5.63\times10^{-2} \pm 1.37\times10^{-2}$
1-Pentanol	71-41-0	$C_5H_{12}O$	6.04	912	$1.93\times10^{-3} \pm 1.70\times10^{-3}$	ND	ND
Linalool	78-70-6	$C_{10}H_{18}O$	11.58	904	$4.64\times10^{-3} \pm 1.60\times10^{-3}$	$1.91\times10^{-3} \pm 2.02\times10^{-3}$	$3.10\times10^{-3} \pm 5.64\times10^{-4}$
α-Terpineol	98-55-5	$C_{10}H_{18}O$	14.94	735	ND	$1.22\times10^{-4} \pm 1.26\times10^{-4}$	ND
**Aldehydes**							
Benzaldehyde	100-52-7	C_7H_6O	11.33	897	$2.02\times10^{-3} \pm 7.53\times10^{-4}$	$1.72\times10^{-3} \pm 1.45\times10^{-3}$	$2.01\times10^{-3} \pm 5.06\times10^{-4}$
Heptanal	111-71-7	$C_7H_{14}O$	5.24	907	$8.81\times10^{-3} \pm 9.74\times10^{-3}$	ND	$9.73\times10^{-3} \pm 5.03\times10^{-3}$
Decanal	112-31-2	$C_{10}H_{20}O$	10.56	829	$2.25\times10^{-3} \pm 2.29\times10^{-3}$	$6.34\times10^{-4} \pm 5.18\times10^{-4}$	$1.80\times10^{-3} \pm 6.18\times10^{-4}$
4-Isopropylbenzaldehyde	122-03-2	$C_{10}H_{12}O$	16.87	950	$0.18 \pm 3.24\times10^{-2}$	$0.14 \pm 7.85\times10^{-2}$	$0.22 \pm 5.74\times10^{-2}$
4-Methoxybenzaldehyde	123-11-5	$C_8H_8O_2$	22.07	781	$1.58\times10^{-3} \pm 2.41\times10^{-3}$	ND	$6.64\times10^{-4} \pm 6.60\times10^{-4}$
Octanal	124-13-0	$C_8H_{16}O$	6.56	951	$1.67\times10^{-2} \pm 1.50\times10^{-2}$	$4.94\times10^{-3} \pm 5.06\times10^{-3}$	$1.64\times10^{-2} \pm 7.61\times10^{-3}$
Nonanal	124-19-6	$C_9H_{18}O$	8.41	936	$5.11\times10^{-2} \pm 3.50\times10^{-2}$	$1.86\times10^{-2} \pm 1.60\times10^{-2}$	$4.68\times10^{-2} \pm 1.60\times10^{-2}$
Tetradecanal	124-25-4	$C_{14}H_{28}O$	24.08	768	$9.89\times10^{-4} \pm 1.51\times10^{-3}$	$6.83\times10^{-4} \pm 4.99\times10^{-4}$	ND
Isovaleraldehyde	590-86-3	$C_5H_{10}O$	2.61	677	ND	ND	$1.77\times10^{-3} \pm 1.74\times10^{-3}$
Hexanal	66-25-1	$C_6H_{12}O$	4.15	918	$3.51\times10^{-2} \pm 3.88\times10^{-2}$	$5.75\times10^{-3} \pm 8.01\times10^{-3}$	$3.55\times10^{-2} \pm 2.25\times10^{-2}$
**Acids**							
Malonamic acid	2345-56-4	$C_3H_5NO_3$	1.65	980	$2.76\times10^{-3} \pm 3.26\times10^{-3}$	$2.47\times10^{-3} \pm 2.65\times10^{-3}$	ND
Ala-gly	687-69-4	$C_5H_{10}N_2O_3$	1.42	981	$1.90\times10^{-2} \pm 1.40\times10^{-2}$	$2.51\times10^{-2} \pm 2.54\times10^{-2}$	$2.71\times10^{-2} \pm 7.93\times10^{-3}$
**Ketones**							
6-Methylhept-5-en-2-one	110-93-0	$C_8H_{14}O$	7.38	847	$9.19\times10^{-4} \pm 6.27\times10^{-4}$	$4.55\times10^{-4} \pm 2.17\times10^{-4}$	$5.71\times10^{-4} \pm 1.37\times10^{-4}$
1,3,3-Trimethylbicyclo [2.2.1] heptan-2-one	1195-79-5	$C_{10}H_{16}O$	8.55	917	$2.87\times10^{-2} \pm 1.96\times10^{-2}$	ND	$1.42\times10^{-2} \pm 1.21\times10^{-2}$
1-(3,5-Di-tert-butyl-4-hydroxy-phenyl)-propan-1-one	14035-34-8	$C_{17}H_{26}O_2$	19.73	747	ND	ND	$4.63\times10^{-4} \pm 4.12\times10^{-4}$
Acetoin	513-86-0	$C_4H_8O_2$	6.85	878	$2.14\times10^{-2} \pm 1.71\times10^{-2}$	$3.30\times10^{-2} \pm 2.56\times10^{-2}$	$1.89\times10^{-2} \pm 1.15\times10^{-2}$
Acetone	67-64-1	C_3H_6O	2.00	769	ND	$1.04\times10^{-2} \pm 8.61\times10^{-3}$	ND

(-)-Fenchone	7787-20-4	$C_{10}H_{16}O$	8.55	949	ND	$1.75\times10^{-2} \pm 1.71\times10^{-2}$	ND
**Ethers**							
Estragole	140-67-0	$C_{10}H_{12}O$	14.38	947	$7.52\times10^{-2} \pm 9.06\times10^{-2}$	$0.11 \pm 8.99\times10^{-2}$	$6.69\times10^{-2} \pm 5.75\times10^{-2}$
Dodecyl octaethylene glycol ether	3055-98-9	$C_{28}H_{58}O_9$	30.32	644	$2.35\times10^{-4} \pm 1.39\times10^{-4}$	$1.52\times10^{-4} \pm 6.70\times10^{-5}$	$4.96\times10^{-4} \pm 3.11\times10^{-4}$
Heptaethylene glycol monododecyl ether	3055-97-8	$C_{26}H_{54}O_8$	31.54	659	$2.42\times10^{-4} \pm 1.60\times10^{-4}$	$2.54\times10^{-4} \pm 1.94\times10^{-4}$	$1.42\times10^{-4} \pm 7.22\times10^{-5}$
Anethole	104-46-1	$C_{10}H_{12}O$	17.87	936	$6.10\times10^{-2} \pm 4.93\times10^{-2}$	$5.26\times10^{-2} \pm 8.17\times10^{-3}$	$3.85\times10^{-2} \pm 2.55\times10^{-2}$
**Hydrocarbons**							
Myrcene	123-35-3	$C_{10}H_{16}$	4.88	880	$1.06\times10^{-2} \pm 9.25\times10^{-3}$	$1.65\times10^{-2} \pm 2.61\times10^{-3}$	$1.20\times10^{-2} \pm 7.26\times10^{-3}$
3-carene	13466-78-9	$C_{10}H_{16}$	4.73	880	$9.52\times10^{-4} \pm 7.95\times10^{-4}$	$1.03\times10^{-3} \pm 2.57\times10^{-4}$	ND
β-pinene	18172-67-3	$C_{10}H_{16}$	4.31	925	$9.85\times10^{-2} \pm 3.57\times10^{-2}$	$5.82\times10^{-2} \pm 5.48\times10^{-2}$	$9.40\times10^{-2} \pm 5.49\times10^{-2}$
p-Menthatriene	18368-95-1	$C_{10}H_{14}$	6.42	768	$2.19\times10^{-3} \pm 1.94\times10^{-3}$	ND	ND
α-Acoradiene	24048-44-0	$C_{15}H_{24}$	14.73	721	$2.36\times10^{-4} \pm 2.24\times10^{-4}$	$3.17\times10^{-4} \pm 1.71\times10^{-4}$	ND
Sabinene	3387-41-5	$C_{10}H_{16}$	4.42	908	$5.07\times10^{-3} \pm 2.82\times10^{-3}$	$6.44\times10^{-3} \pm 1.09\times10^{-3}$	$3.85\times10^{-3} \pm 3.42\times10^{-3}$
2,4-Thujadiene	36262-09-6	$C_{10}H_{14}$	4.55	719	$5.77\times10^{-4} \pm 4.71\times10^{-4}$	$7.07\times10^{-4} \pm 6.15\times10^{-4}$	ND
(E)-3,7-Dimethylocta-1,3,6-triene	3779-61-1	$C_{10}H_{16}$	5.71	847	$4.64\times10^{-3} \pm 2.42\times10^{-3}$	$5.57\times10^{-3} \pm 8.13\times10^{-4}$	$4.70\times10^{-3} \pm 1.16\times10^{-3}$
β-Phellandrene	555-10-2	$C_{10}H_{16}$	4.42	878	$9.66\times10^{-3} \pm 6.04\times10^{-3}$	$6.92\times10^{-3} \pm 5.94\times10^{-3}$	$5.65\times10^{-3} \pm 6.04\times10^{-3}$
D-Limonene	5989-27-5	$C_{10}H_{16}$	5.31	918	$8.65\times10^{-2} \pm 1.61\times10^{-2}$	$8.14\times10^{-2} \pm 1.21\times10^{-2}$	$4.58\times10^{-2} \pm 4.05\times10^{-2}$
(+)-α-Pinene	7785-70-8	$C_{10}H_{16}$	3.50	920	$1.37\times10^{-2} \pm 2.15\times10^{-3}$	$1.34\times10^{-2} \pm 2.09\times10^{-3}$	$1.46\times10^{-2} \pm 3.32\times10^{-3}$
Camphene	79-92-5	$C_{10}H_{16}$	3.96	908	$7.75\times10^{-4} \pm 6.37\times10^{-4}$	$8.68\times10^{-4} \pm 4.74\times10^{-4}$	$9.40\times10^{-4} \pm 5.31\times10^{-4}$
γ-Terpinene	99-85-4	$C_{10}H_{16}$	5.90	934	$0.17 \pm 5.29\times10^{-2}$	$0.18 \pm 2.60\times10^{-2}$	$8.76\times10^{-2} \pm 7.16\times10^{-2}$
Methylbenzene	108-88-3	C_7H_8	3.75	786	$7.52\times10^{-4} \pm 5.55\times10^{-4}$	$3.10\times10^{-3} \pm 1.99\times10^{-3}$	$8.75\times10^{-4} \pm 6.44\times10^{-4}$
p-Cymene	99-87-6	$C_{10}H_{14}$	6.31	929	ND	0.14 ± 0.11	ND
α-Fenchene	471-84-1	$C_{10}H_{16}$	3.88	791	ND	$1.85\times10^{-4} \pm 1.59\times10^{-4}$	ND

M-Cymene	535-77-3	$C_{10}H_{14}$	6.31	918	ND	ND	0.13 ± 0.11
4-Isopropenyltoluene	1195-32-0	$C_{10}H_{12}$	9.33	734	$3.00\times10^{-4} \pm 2.46\times10^{-4}$	$2.69\times10^{-4} \pm 1.70\times10^{-4}$	ND
Octane	111-65-9	C_8H_{18}	1.88	842	$1.56\times10^{-3} \pm 1.77\times10^{-3}$	$4.18\times10^{-3} \pm 4.14\times10^{-3}$	$1.65\times10^{-3} \pm 3.84\times10^{-4}$
α-Terpinene	99-86-5	$C_{10}H_{16}$	5.09	847	$2.52\times10^{-3} \pm 1.29\times10^{-3}$	$2.99\times10^{-3} \pm 7.20\times10^{-4}$	$3.19\times10^{-3} \pm 5.53\times10^{-4}$
**Others**							
Eucalyptol	470-82-6	$C_{10}H_{18}O$	5.49	806	ND	ND	$7.61\times10^{-3} \pm 6.39\times10^{-3}$
Pinocarveol, trans- (-)-	547-61-5	$C_{10}H_{16}O$	14.05	694	ND	$2.31\times10^{-4} \pm 2.35\times10^{-4}$	ND
Phellandral	21391-98-0	$C_{10}H_{16}O$	15.53	663	ND	$5.03\times10^{-4} \pm 1.07\times10^{-4}$	$6.45\times10^{-4} \pm 1.46\times10^{-4}$
1-Methoxyhexane	4747-07-3	$C_7H_{16}O$	7.63	842	$4.53\times10^{-3} \pm 2.98\times10^{-3}$	$2.18\times10^{-3} \pm 1.66\times10^{-3}$	ND

RT: Retention index calculated for TG-WAXMS B capillary column (30 m × 0.25 mm × 0.25 μm). ND: not detected.

Table S3. The relative content of volatile compounds in yak jerky from different muscles characterized by GC-IMS (Mean ± SD).

Compounds	CAS	Formula	MW *	RI	RT (s)	DT (ms)	Relative content		
							TB	LT	BF
**Aldehydes**									
Hexanal-D	66-25-1	$C_6H_{12}O$	100.2	1098.1	462.41	1.55571	$8.18\times10^{-2} \pm 2.39\times10^{-2\,a}$	$3.31\times10^{-2} \pm 2.00\times10^{-3\,b}$	$8.62\times10^{-2} \pm 2.08\times10^{-2\,a}$
Hexanal-M	66-25-1	$C_6H_{12}O$	100.2	1098.4	463.021	1.26874	$0.17 \pm 2.03\times10^{-2\,a}$	$0.10 \pm 4.17\times10^{-3\,b}$	$0.16 \pm 1.04\times10^{-2\,a}$
Pentanal	110-62-3	$C_5H_{10}O$	86.1	999.4	347.782	1.41982	$2.36\times10^{-2} \pm 3.55\times10^{-3\,a}$	$2.99\times10^{-2} \pm 3.06\times10^{-3\,a}$	$1.94\times10^{-2} \pm 1.13\times10^{-3\,a}$
2-Methyl propanal-D	78-84-2	C_4H_8O	72.1	856.2	263.649	1.27971	$7.31\times10^{-3} \pm 7.01\times10^{-4\,a}$	$4.46\times10^{-3} \pm 1.72\times10^{-4\,b}$	$7.26\times10^{-3} \pm 4.39\times10^{-4\,a}$
2-Methyl propanal-M	78-84-2	C_4H_8O	72.1	830.8	249.054	1.09949	$2.62\times10^{-3} \pm 1.48\times10^{-4\,b}$	$2.76\times10^{-3} \pm 7.41\times10^{-5\,b}$	$3.44\times10^{-3} \pm 5.05\times10^{-5\,a}$
Heptanal	111-71-7	$C_7H_{14}O$	114.2	1193.3	678.713	1.34037	$5.68\times10^{-2} \pm 6.49\times10^{-3\,a}$	$5.23\times10^{-2} \pm 2.17\times10^{-3\,a}$	$6.15\times10^{-2} \pm 3.49\times10^{-3\,a}$
3-Methylbutanal-D	590-86-3	$C_5H_{10}O$	86.1	903.5	290.772	1.19969	$3.57\times10^{-2} \pm 6.19\times10^{-3\,a}$	$4.47\times10^{-2} \pm 1.72\times10^{-3\,a}$	$3.76\times10^{-2} \pm 2.67\times10^{-3\,a}$
3-Methylbutanal-M	590-86-3	$C_5H_{10}O$	86.1	919.1	299.737	1.17032	$1.32\times10^{-2} \pm 1.49\times10^{-3\,a}$	$1.72\times10^{-2} \pm 9.94\times10^{-4\,a}$	$1.64\times10^{-2} \pm 6.77\times10^{-4\,a}$

Diethyl acetal	105-57-7	$C_6H_{14}O_2$	118.2	894.9	285.815	1.13061	$3.07\times10^{-2} \pm 3.73\times10^{-3\,a}$	$3.53\times10^{-2} \pm 1.75\times10^{-3\,a}$	$3.51\times10^{-2} \pm 1.53\times10^{-3\,a}$
**Ketones**									
Cyclohexanone	108-94-1	$C_6H_{10}O$	98.1	1293.0	1011.688	1.1625	$1.23\times10^{-2} \pm 8.36\times10^{-4\,a}$	$8.36\times10^{-3} \pm 5.35\times10^{-4\,a}$	$1.01\times10^{-2} \pm 7.35\times10^{-4\,a}$
Propan-2-one-D	67-64-1	C_3H_6O	58.1	847.8	258.8	1.14608	$3.82\times10^{-2} \pm 7.90\times10^{-3\,a}$	$1.88\times10^{-2} \pm 1.24\times10^{-3\,b}$	$3.99\times10^{-2} \pm 6.08\times10^{-3\,a}$
Propan-2-one-M	67-64-1	C_3H_6O	58.1	861.0	266.386	1.114	$0.30 \pm 1.07\times10^{-2\,a}$	$0.30 \pm 1.32\times10^{-2\,a}$	$0.30 \pm 1.13\times10^{-2\,a}$
2-Butanone	78-93-3	C_4H_8O	72.1	864.0	268.102	1.06156	$5.91\times10^{-3} \pm 6.80\times10^{-4\,a}$	$5.95\times10^{-3} \pm 1.95\times10^{-4\,a}$	$7.01\times10^{-3} \pm 3.96\times10^{-4\,a}$
Cyclopentanone	120-92-3	C_5H_8O	84.1	1212.2	741.932	1.33696	$1.29\times10^{-2} \pm 2.07\times10^{-3\,a}$	$1.58\times10^{-2} \pm 1.24\times10^{-3\,a}$	$1.40\times10^{-2} \pm 1.40\times10^{-3\,a}$
3-Hydroxy-2-butanone-D	513-86-0	$C_4H_8O_2$	88.1	1292.0	1008.407	1.32793	$4.50\times10^{-2} \pm 5.49\times10^{-3\,a}$	$2.98\times10^{-2} \pm 2.05\times10^{-3\,a}$	$6.26\times10^{-2} \pm 1.13\times10^{-2\,a}$
3-Hydroxy-2-butanone-M	513-86-0	$C_4H_8O_2$	88.1	1296.8	1023.798	1.07025	$0.45 \pm 1.69\times10^{-2\,ab}$	$0.36 \pm 1.48\times10^{-2\,b}$	$5.04\times10^{-1} \pm 3.51\times10^{-2\,a}$
Hexan-2-one	591-78-6	$C_6H_{12}O$	100.2	1100.0	466.685	1.47855	$2.38\times10^{-2} \pm 3.00\times10^{-3\,a}$	$1.59\times10^{-2} \pm 6.52\times10^{-4\,a}$	$2.43\times10^{-2} \pm 2.73\times10^{-3\,a}$
Butan-2-one-D	78-93-3	C_4H_8O	72.1	929.8	305.866	1.26874	$3.83\times10^{-2} \pm 3.26\times10^{-3\,a}$	$3.61\times10^{-2} \pm 1.85\times10^{-3\,a}$	$4.19\times10^{-2} \pm 2.69\times10^{-3\,a}$
Butan-2-one-M	78-93-3	C_4H_8O	72.1	915.2	297.506	1.24177	$9.90\times10^{-3} \pm 7.35\times10^{-4\,a}$	$1.50\times10^{-2} \pm 1.92\times10^{-3\,a}$	$1.26\times10^{-2} \pm 1.44\times10^{-3\,a}$
**Alcohols**									
3-Methylbutan-1-ol-D	123-51-3	$C_5H_{12}O$	88.1	1213.6	746.718	1.24596	$5.20\times10^{-2} \pm 9.07\times10^{-3\,a}$	$7.05\times10^{-2} \pm 6.25\times10^{-3\,a}$	$5.75\times10^{-2} \pm 8.72\times10^{-3\,a}$
3-Methylbutan-1-ol-M	123-51-3	$C_5H_{12}O$	88.1	1212.7	743.551	1.49016	$1.21\times10^{-2} \pm 9.09\times10^{-4\,a}$	$1.50\times10^{-2} \pm 1.49\times10^{-3\,a}$	$1.31\times10^{-2} \pm 1.44\times10^{-3\,a}$
2-Methyl-1-propanol	78-83-1	$C_4H_{10}O$	74.1	1101.7	470.423	1.17282	$4.60\times10^{-2} \pm 5.71\times10^{-3\,a}$	$4.87\times10^{-2} \pm 2.62\times10^{-3\,a}$	$4.44\times10^{-2} \pm 4.45\times10^{-3\,a}$
Ethanol	64-17-5	C_2H_6O	46.1	922.2	301.493	1.04092	$2.57\times10^{-2} \pm 5.53\times10^{-3\,a}$	$3.44\times10^{-2} \pm 2.19\times10^{-3\,a}$	$3.57\times10^{-2} \pm 1.97\times10^{-3\,a}$
1,8-Cineole	616-25-1	$C_{10}H_{18}O$	154.3	1204.4	715.922	1.29636	$1.76\times10^{-2} \pm 2.72\times10^{-3\,a}$	$1.59\times10^{-2} \pm 5.82\times10^{-4\,a}$	$1.83\times10^{-2} \pm 2.26\times10^{-3\,a}$
1-Penten-3-ol	71-41-0	$C_5H_{10}O$	86.1	1111.4	492.392	1.33956	$1.59\times10^{-2} \pm 1.40\times10^{-3\,a}$	$1.64\times10^{-2} \pm 1.50\times10^{-3\,a}$	$2.00\times10^{-2} \pm 7.82\times10^{-4\,a}$
1-Pentanol	123-51-3	$C_5H_{12}O$	88.1	1261.4	906.254	1.2508	$1.59\times10^{-2} \pm 1.4\times10^{-3\,b}$	$3.21\times10^{-2} \pm 1.91\times10^{-3\,a}$	$2.03\times10^{-2} \pm 1.79\times10^{-3\,b}$
**Esters**									
Butyl pentanoate	591-68-4	$C_9H_{18}O_2$	158.2	1296.0	1021.221	1.40887	$2.37\times10^{-2} \pm 1.81\times10^{-3\,a}$	$2.59\times10^{-2} \pm 1.67\times10^{-3\,a}$	$2.59\times10^{-2} \pm 1.62\times10^{-3\,a}$

1-methylethyl acetate	108-21-4	$C_5H_{10}O_2$	102.1	852.0	261.239	1.47059	$6.55\times10^{-3} \pm 1.97\times10^{-4}$ [a]	$5.10\times10^{-3} \pm 2.01\times10^{-4}$ [b]	$6.17\times10^{-3} \pm 3.23\times10^{-4}$ [ab]
Ethyl crotonate	623-70-1	$C_6H_{10}O_2$	114.1	1147.6	574.152	1.17678	$1.96\times10^{-2} \pm 1.44\times10^{-3}$ [a]	$1.51\times10^{-2} \pm 1.00\times10^{-3}$ [a]	$2.01\times10^{-2} \pm 1.02\times10^{-3}$ [a]
Hexyl butyrate	2639-63-6	$C_{10}H_{20}O_2$	172.3	1401.2	1336.676	1.48151	$0.11 \pm 5.56\times10^{-3}$ [a]	$0.10 \pm 1.52\times10^{-3}$ [a]	$0.11 \pm 2.24\times10^{-3}$ [a]
Ethyl butanoate	105-54-4	$C_6H_{12}O_2$	116.2	1046.1	401.397	1.19723	$1.27\times10^{-2} \pm 1.59\times10^{-3}$ [a]	$1.25\times10^{-2} \pm 8.40\times10^{-4}$ [a]	$1.75\times10^{-2} \pm 1.85\times10^{-3}$ [a]
Ethyl Acetate-D	141-78-6	$C_4H_8O_2$	88.1	903.2	290.608	1.33535	$0.14 \pm 3.68\times10^{-2}$ [a]	$0.15 \pm 5.13\times10^{-3}$ [a]	$8.31\times10^{-2} \pm 1.45\times10^{-2}$ [a]
Ethyl Acetate-M	141-78-6	$C_4H_8O_2$	88.1	905.0	291.609	1.09436	$9.36\times10^{-2} \pm 1.51\times10^{-2}$ [a]	$9.79\times10^{-2} \pm 1.02\times10^{-3}$ [a]	$8.25\times10^{-2} \pm 7.80\times10^{-3}$ [a]
Ethyl formate	109-94-4	$C_3H_6O_2$	74.1	845.2	257.321	1.05991	$7.71\times10^{-2} \pm 3.80\times10^{-3}$ [a]	$5.49\times10^{-2} \pm 1.61\times10^{-3}$ [b]	$7.71\times10^{-2} \pm 2.04\times10^{-3}$ [a]
Methyl acetate	79-20-9	$C_3H_6O_2$	74.1	857.0	264.099	1.19685	$1.77\times10^{-2} \pm 9.61\times10^{-4}$ [a]	$1.22\times10^{-2} \pm 4.16\times10^{-4}$ [b]	$1.66\times10^{-2} \pm 6.99\times10^{-4}$ [a]
**Hydrocarbons**									
γ-Terpinene	99-85-4	$C_{10}H_{16}$	136.2	1246.0	854.843	1.21336	$7.22\times10^{-2} \pm 2.35\times10^{-2}$ [a]	$6.23\times10^{-2} \pm 9.32\times10^{-3}$ [a]	$6.64\times10^{-2} \pm 6.69\times10^{-3}$ [a]
α-Pinene	80-56-8	$C_{10}H_{16}$	136.2	999.0	347.288	1.29384	$6.40\times10^{-2} \pm 9.91\times10^{-3}$ [b]	$0.11 \pm 7.71\times10^{-3}$ [a]	$7.32\times10^{-2} \pm 5.38\times10^{-3}$ [ab]
Limonene-D	138-86-3	$C_{10}H_{16}$	136.2	1200.8	703.746	1.29215	$1.00\times10^{-2} \pm 1.99\times10^{-3}$ [a]	$7.18\times10^{-3} \pm 7.77\times10^{-4}$ [a]	$8.65\times10^{-3} \pm 5.69\times10^{-4}$ [a]
Limonene-M	138-86-3	$C_{10}H_{16}$	136.2	1199.5	699.618	1.21366	$9.24\times10^{-3} \pm 2.05\times10^{-3}$ [a]	$6.78\times10^{-3} \pm 6.21\times10^{-4}$ [a]	$7.77\times10^{-3} \pm 6.90\times10^{-4}$ [a]
α-Terpinolene	586-62-9	$C_{10}H_{16}$	136.2	1292.3	1009.319	1.20507	$2.93\times10^{-2} \pm 3.85\times10^{-3}$ [a]	$2.70\times10^{-2} \pm 1.55\times10^{-3}$ [a]	$3.24\times10^{-2} \pm 9.65\times10^{-4}$ [a]
β-Pinene-D	127-91-3	$C_{10}H_{16}$	136.2	1111.6	492.938	1.29213	$3.39\times10^{-2} \pm 1.05\times10^{-2}$ [a]	$2.83\times10^{-2} \pm 3.85\times10^{-3}$ [a]	$3.56\times10^{-2} \pm 2.78\times10^{-3}$ [a]
β-Pinene-M	127-91-3	$C_{10}H_{16}$	136.2	1113.3	496.766	1.21357	$6.09\times10^{-2} \pm 1.67\times10^{-2}$ [a]	$5.83\times10^{-2} \pm 8.24\times10^{-3}$ [a]	$7.04\times10^{-2} \pm 5.23\times10^{-3}$ [a]
α-Terpinene	1120-21-4	$C_{10}H_{16}$	136.2	1165.2	613.947	1.21262	$6.09\times10^{-3} \pm 1.18\times10^{-3}$ [a]	$5.10\times10^{-3} \pm 4.19\times10^{-4}$ [a]	$5.62\times10^{-3} \pm 3.97\times10^{-4}$ [a]
**Ethers**									
1,2-Dimethoxyethane	110-71-4	$C_4H_{10}O_2$	90.1	934.6	308.613	1.31132	$2.74\times10^{-2} \pm 1.49\times10^{-3}$ [a]	$2.20\times10^{-2} \pm 8.15\times10^{-4}$ [b]	$2.83\times10^{-2} \pm 1.39\times10^{-3}$ [a]
**Others**									
Dimethyl trisulfide	3658-80-8	$C_2H_6S_3$	126.3	1383.0	1282.104	1.30592	$3.79\times10^{-2} \pm 6.49\times10^{-3}$ [a]	$3.57\times10^{-2} \pm 1.03\times10^{-3}$ [a]	$4.06\times10^{-2} \pm 5.93\times10^{-3}$ [a]
2,6-Dimethylpyrazine	108-50-9	$C_6H_8N_2$	108.1	1352.4	1190.372	1.14054	$1.17\times10^{-2} \pm 2.27\times10^{-3}$ [a]	$1.71\times10^{-2} \pm 1.14\times10^{-3}$ [a]	$1.02\times10^{-2} \pm 1.01\times10^{-3}$ [a]
Undecane	470-82-6	$C_{11}H_{24}$	156.3	1098.4	463.058	1.36348	$0.12 \pm 1.32\times10^{-2}$ [a]	$8.71\times10^{-2} \pm 2.25\times10^{-3}$ [a]	$0.12 \pm 1.20\times10^{-2}$ [a]

* MW, RI, RT and DT stand for volatile compounds mass, retention index, retention time, and drift time, respectively. For each volatile compound, SEM values followed by a common superscript identify no significant differences.

Table S4. ROAV of volatile compounds in yak jerky from different muscles characterized by GC-MS and GC-IMS.

	Compound Name	Threshold (μg/kg)	ROAV		
			TB	LT	BF
GC-MS	1-Nonanol	0.086	–	15.26333	–
	1-Octen-3-ol	1.5	4.708299	9.998937	–
	Linalool	0.22	45.38041	48.17301	33.12655
	Eucalyptol	3	–	–	5.961556
	Heptanal	3	6.32326	–	7.616767
	Decanal	3	1.616609	1.172509	1.407155
	Octanal	0.8	44.82434	34.27703	48.30268
	Nonanal	1.1	100	93.77135	100
	Isovaleraldehyde	0.25	–	–	16.63372
	Hexanal	5	15.10421	6.376759	16.69728
	Acetoin	55	0.83823	3.325057	0.807101
	Estragole	6	26.9973	100	26.19999
	Anethole	100	1.312637	2.918049	0.903481
	3-Carene	0.4	5.123714	14.24697	–
	β-Pinene	180	1.177804	1.793961	1.226331
	β-Phellandrene	8	2.600234	4.797697	1.658129
	D-Limonene	10	18.62289	45.15313	10.75404
	γ-Terpinene	260	1.425817	3.924652	0.791342
	p-Cymene	150	–	5.132583	–

GC-IMS	Hexanal-D	5	1.831927	0.929906	1.673773
	Hexanal-M	5	3.779587	2.878009	3.201198
	Heptanal	3	2.102121	2.445706	2.035858
	3-Methylbutanal-D	2	1.963626	3.126661	1.89134
	3-Methylbutanal-M	2	0.727509	1.20218	0.81688
	3-Hydroxy-2-butanone-D	0.5	9.817304	8.330039	12.23155
	3-Hydroxy-2-butanone-M	0.5	100	100	100
	1,8-Cineole	1.1	1.767917	2.027992	1.668768
	1-Penten-3-ol	1	1.763408	2.296541	1.994543
	Ethyl acetate-D	5	3.138101	4.167997	1.689476
	Ethyl acetate-M	5	2.072687	2.745426	1.663301
	Ethyl formate	5	1.707558	1.541432	1.534396
	Dimethyl trisulfide	1	4.195834	5.020411	4.079195

"–" means not detected.
